# Psychometric properties of an instrument for assessing the experience of patients treated with inhaled insulin: the Inhaled Insulin Treatment Questionnaire (IITQ)

**DOI:** 10.1186/1477-7525-8-32

**Published:** 2010-03-24

**Authors:** Richard R Rubin, Mark Peyrot

**Affiliations:** 1 Department of Medicine, The Johns Hopkins University School of Medicine, 946 East Piney Hill Road, Monkton, Baltimore, Maryland, MD 21111, USA; 2 Department of Pediatrics, The Johns Hopkins University School of Medicine, Baltimore, Maryland, USA; 3 Department of Sociology, Loyola University Maryland, Baltimore, Maryland, USA

## Abstract

**Background:**

Along with general measures of treatment satisfaction, treatment-specific and device-specific treatment satisfaction should be assessed in clinical trials, because these latter measures may be more strongly correlated with clinical outcomes.

**Methods:**

Study participants were 1076 adults (type 1 = 509, type 2 = 567) in clinical trials of Technosphere Insulin^®^, who completed the SF-36 health-related quality of life questionnaire and the Inhaled Insulin Treatment Questionnaire (IITQ), a new instrument assessing diabetes worries, perceptions of insulin therapy, treatment satisfaction, treatment preference, and inhaler performance. The IITQ was administered twice prior to treatment initiation in the clinical trials, 1-2 weeks apart, and several times during the trials. Inhaler performance was assessed at follow-up visits, after participants had used the device.

**Results:**

IITQ subscales had acceptable reliability (alpha = 0.68-0.87, median 0.83) and test-retest correlations (intra-class correlation coefficient = 0.67-0.90, median 0.82); floor effects (0.2-2.8%) and ceiling effects (0-9.3%) were minimal. Reliabilities for inhaler performance measures were acceptable (alpha = 0.73-0.90, median 0.85); there were no floor effects (0.0%) and ceiling effects (4.9-39.0%) were moderate. There were several modest associations between IITQ scores and measures of health status. Diabetes worries were lower for participants who had better mental health (type 2) and for those with higher BMI; perceptions of insulin therapy were more favorable for participants who had better physical and mental health; treatment satisfaction was higher for patients who had lower BMI (type 2), lower A1c levels, and better physical health (type 2); treatment preference was higher for patients with lower BMI (type 2) and better mental health (type 1).

**Conclusions -:**

Preliminary findings suggest that the IITQ is a comprehensive, reliable measure of the experience of patients treated with inhaled insulin.

## Background

Patient-reported outcomes (PRO) are now accepted as important outcomes in assessing the effects of new therapies because PRO may affect treatment adherence [1] and consequent clinical outcomes [2-4]. An instrument for assessing PRO is valuable to the extent it reliably, validly, and comprehensively assesses the benefits and burdens of a specific therapy. A comprehensive assessment of PRO should include measures of general concerns (e.g., diabetes-related worries), treatment-specific measures (e.g., perceptions of insulin therapy), and treatment satisfaction and treatment preference, as well as device-specific measures when applicable (e.g., insulin delivery system perceptions). Comprehensive diabetes treatment satisfaction instruments include the Insulin Delivery System Rating Questionnaire (IDSRQ) [5] and the Pramlintide Treatment Satisfaction Questionnaire (PRAM-TSQ) [6].

Treatment-specific and device-specific measures should be included in assessment instruments because they may be more strongly correlated with clinical outcomes than more general diabetes treatment satisfaction measures [7]. Thus more specific measures may be more responsive to differences among treatments. For example, a question such as "how much does your insulin delivery system interfere with your ability to get a good night's sleep?" (from the IDSRQ), is likely to be more sensitive to differences among insulin delivery systems than a question such as "how satisfied are you with your understanding of diabetes? (from the Diabetes Treatment Satisfaction Questionnaire) [8].

The Inhaled Insulin Treatment Questionnaire (IITQ) was developed to meet the goal of creating a single questionnaire that assessed the broad range of patient-reported outcomes relevant to use of inhaled insulin. These include more general issues such as general diabetes worries, overall treatment satisfaction, and treatment preference - all of which could apply to any diabetes therapy and could potentially discriminate among therapies, perceptions of insulin convenience, ease of use, facilitation of self-care, and clinical efficacy - which could apply to any insulin therapy and potentially discriminate inhaled and injected insulin therapy, and perceptions of the device used to deliver inhaled insulin that could apply only to an inhaled insulin delivery system. Thus the IITQ incorporates elements that allow for comparisons among all diabetes therapies, among all insulin therapies, and among all inhaled insulin therapies.

The IITQ was used in clinical trials of Technosphere Insulin^®^. The Technosphere Insulin^® ^(TI) system, a proprietary product of MannKind Corporation, consists of a dry powder formulation of monomer human (rDNA origin) mealtime insulin that is inhaled into the deep lung using the MedTone Inhaler^®^, a pocket-sized, passive, breath-powered device [9-11]. In a previous randomized, controlled trial, using an earlier version of the IITQ (the ITQ), insulin naïve individuals with type 2 diabetes were randomized to active TI or placebo TI therapy at mealtimes [12]. Perceptions of insulin therapy improved significantly during the trial in the active TI arm (effect size for overall measure = .56, p = 0.002) but not in the placebo arm, with no significant difference between arms. The majority of subjects rated inhaler performance positively (median = 93% positive ratings).

The current study, which includes patients with type 1 diabetes and patients with type 2 diabetes, was designed to assess the psychometric properties of the IITQ, including: 1) dimensionality (i.e., subscales), 2) inter-item agreement, 3) test-retest reliability, 4) floor/ceiling effects, and 5) association of IITQ measures with study participant characteristics.

## Methods

### Subjects and study protocol

Subjects providing data for this paper were drawn from two multi-center, multi-national, 45-week studies conducted by MannKind Corporation. Subjects in one study were adults with type 1 diabetes randomized to mealtime treatment with either TI or rapid-acting insulin aspart as an adjunct to basal insulin therapy. Subjects in the second study were adults with type 2 diabetes randomized to treatment with mealtime TI 2-3 times a day and basal insulin, or treatment with biphasic insulin aspart 70/30.

Inclusion criteria for both studies were: HbA_1c _>7.0% and ≥ 11.0%, non-smoking, Forced Expiratory Volume in 1 Second (FEV_1_) ≥ 70% predicted, Carbon monoxide diffusing capacity (DLco) ≥ 80% predicted, Total Lung Capacity (TLC) ≥ 80% predicted. Exclusion criteria for all subjects were: history of viral and/or cirrhotic hepatic disease and/or abnormal liver enzymes, history of chronic obstructive pulmonary disease (COPD), asthma, and/or any other clinically important pulmonary disease, or evidence of severe complications of diabetes. Additional inclusion criteria for subjects with type 1 diabetes were: current use of subcutaneous (sc) basal/prandial insulin therapy, and BMI ≤ 35 kg/m^2^. Additional inclusion criteria for subjects with type 2 diabetes were: BMI ≤ 40 kg/m^2^, and current use of sc insulin more than once daily (with or without concomitant metformin or thiazolidinediones). Additional exclusion criteria for subjects with type 2 diabetes include use of sulfonylureas, meglitindes, alpha glucosidase inhibitors, pramlintide acetate, and/or incretins within the preceding 8 weeks.

Both studies complied with the Declaration of Helsinki for participation in human research and received appropriate institutional review board approvals prior to initiation. All study participants gave written informed consent before entering into the studies.

Subjects who provided consent, provided baseline data, and completed the PRO measures were included in the analyses reported here. Only those subjects who answered all IITQ questions at both pre-treatment administrations were included in this study.

### Questionnaire development

The IITQ assesses: Diabetes Worries (5 questions), Perceptions of Insulin Therapy (16 questions), Treatment System Satisfaction (3 items), Treatment System Preference (1 item) and Inhaler Performance (10 questions). The IITQ appears in Additional file 1 (scoring instructions appear in Additional file 2). An earlier version of the IITQ (the ITQ) included only 12 items assessing perceptions of insulin therapy, and no items assessing treatment system satisfaction, or treatment system preference. Additional items in the current version of the IITQ are: several perceptions of insulin therapy (difficulty taking insulin at the right time, and effect of insulin on overall glucose control, on high glucose levels, and on low glucose levels); treatment system satisfaction (overall satisfaction, willingness to continue using the system, willingness to recommend the system to others); treatment system preference (preference for the system compared to the one previously used). Several items measuring inhaler performance in the earlier version were dropped from this version in order to make it more broadly applicable to alternative inhaled insulin devices.

The IITQ subscales assessing general diabetes worries, perceptions of clinical efficacy, overall treatment satisfaction, and treatment preference (i.e., all measures except for perceptions of the insulin inhaler), were based on the validated Insulin Delivery System Rating Questionnaire (IDSRQ) [5]. The IDSRQ was developed through a procedure in which focus groups were asked about the advantages and disadvantages of the various insulin delivery systems they used. Content validity of the IITQ was assessed by cognitive debriefing methods, to determine whether patients understand concepts and items in the same way that the instrument's developers intended. The results of this effort, involving 15 patients with type 1 or type 2 diabetes, indicated very few difficulties with the IITQ, and no more than a small minority of study participants mentioned any given difficulty.

The IITQ was translated from English into the languages of each country in which each study was conducted. IITQ translations were then back-translated into English and these back-translations were reviewed by the authors of this paper. When the back- translation differed from the original English version of the IITQ the authors discussed these discrepancies with the translators in order to arrive at an IITQ translation that was as close as possible to the meaning of the original English version.

### Measures

In addition to a variety of clinical outcome measures, participants in both studies completed the SF-36. This instrument consists of 36 questions that generate two composite scores, the Physical Component Summary (PCS) and the Mental Component Summary (MCS). QualityMetric performed the scoring analysis to generate scores ranging from 0 to 100, with higher scores indicating better quality of life. The QualityMetric Missing Data Estimation algorithms were used to score all SF-36 measures [13].

The IITQ was administered twice prior to treatment, approximately 1-2 weeks apart, to assess test-retest reliability. The IITQ was re-administered later in the study, but for the most part, follow-up data are not analyzed here. However, items specific to Inhaler Performance could not be asked until the follow-up visits, so data regarding these items are derived from the initial follow-up administration of the IITQ. IITQ responses were obtained during regularly scheduled visits, with standardized timing and conditions of administration.

The IITQ assesses diabetes-specific, treatment-specific, and device-specific, patient-reported outcomes in patients using inhaled insulin. The IITQ differs from other instruments designed to assess PRO associated with inhaled insulin treatment [14,15]. The IITQ covers a broad range of outcomes, as do comprehensive instruments [16], but is shorter and contains items specific to inhaled insulin.

Different versions of the IITQ were used pre-treatment and at follow-up. The pre-treatment version did not include inhaler perception items (items 26-35 in Additional file 1) because participants would not have used the inhaler at that time. Study participants randomized to TI completed the entire questionnaire (items 1-35) at follow-up visits; study participants randomized to rapid-acting insulin aspart (type 1 diabetes study) or to biphasic insulin aspart (type 2 diabetes study) completed the same version of the IITQ at baseline and follow-up visits (items 1-25). All IITQ items had a 6-point response scale (strongly disagree = 0, disagree = 20, mildly disagree = 40, mildly agree = 60, agree = 80, strongly agree = 100). Where necessary, items were reverse scored so that all were in the direction of greater worries, more positive perceptions of insulin therapy, higher treatment satisfaction and preference, and more positive perceptions of inhaler performance. IITQ items from each of the domains were combined into composite additive scales for analysis.

### Statistical analysis

Descriptive statistics (percentages, means, standard deviations) are presented for participant characteristics at baseline. Several psychometric analyses were performed for the IITQ. Factor analyses were performed on all IITQ domains with more than one item: Diabetes Worries, Perceptions of Insulin Therapy, Treatment Satisfaction and Inhaler Performance. Reliability (inter-item agreement) was assessed by Cronbach's alpha for both of the baseline administrations. Test-retest reliability was assessed by the intraclass correlation coefficient and paired t-tests for difference in means. Pearson correlations were used to estimate associations of IITQ scores from the initial administration with baseline subject characteristics (sex, race/ethnicity, age, weight, BMI, A1c, duration of diabetes) and baseline SF-36 scores. We did not expect strong correlations because there is no hypothesized causal connection between the scales and most correlates. We did expect better health status, including SF-36 scores, to be associated with lower IITQ worries scores and higher IITQ treatment satisfaction scores. We expected low correlations between IITQ subscale scores and other correlates; this would indicate that IITQ responses are not heavily influenced by extraneous factors, making the IITQ appropriate for multiple populations. Psychometric analyses were performed by combining subjects in the trial treatment arms; separate analyses were performed for the two study populations.

Minimum levels of statistical significance for all analyses were set at p < 0.05, two-tailed. All analyses were conducted using SPSS 14.

## Results

Study participants (Table 1) were 509 patients with type 1 diabetes and 567 participants with type 2 diabetes. Participants were approximately evenly divided by gender, and the majority was non-Hispanic White (84.5% of type 1 and 66.0% of type 2). The mean age differed substantially between type 1 (37.4 years) and type 2 (55.4 years) populations. Mean duration of diabetes also varied with study population (18.4 years for type 1 and 13.1 years for type 2). Type 2 patients were heavier than type 1 participants (BMI = 31.4 kg/m^2 ^vs. 26.1 kg/m^2^). Glucose control was similar for type 1 and type 2 (A1c = 8.4% and 8.7%, respectively).

**Table 1:** Study Population Baseline Characteristics

Characteristic	Type 1 Study (n = 509)	Type 2 Study (n = 567)
**Gender, N (%)**		

Male	267 (52.5)	271 (47.8)

Female	242 (47.5)	296 (52.2)

**Race, N (%)**		

Non-Hispanic White	430 (84.5)	374 (66.0)

African American	33 (6.5)	49 (8.6)

Asian American	6 (1.2)	11 (1.9)

Hispanic	33 (6.5)	121 (21.3)

Other	7 (1.4)	12 (2.1)

**Age, Years**		

Mean (SD)	37.4 (13.0)	55.4 (10.2)

**Duration of Diabetes, Years**		

Mean (SD)	18.4 (11.6)	13.1 (7.5)

**Weight, Kilograms**		

Mean (SD)	76.6 (15.1)	87.8 (17.7)

**BMI, kg/m2**		

Mean (SD)	26.1 (3.8)	31.4 (4.8)

**HbA1c (%)**		

Mean (SD)	8.4 (1.0)	8.7 (1.1)

**SF-36 PCS**		

Mean (SD)	53.3 (6.5)	46.7 (9.2)

**SF-36 MCS**		

Mean (SD)	50.3 (9.0)	49.2 (10.9)

Factor analysis (results not shown) of items from the Diabetes Worries and Treatment Satisfaction domains revealed that each set of items formed only one factor; therefore only a single score was generated for each of these domains. Items from the Perceptions of Insulin Therapy domain formed two distinct factors, one representing positively worded items and the other negatively worded items. Because of the lack of substantive meaning for the separate factors, only a single composite scale was generated. Items from the Inhaler Performance domain formed three distinct factors, one regarding the device (5 items), another regarding the cartridge (3 items), and a third regarding dosing (2 items); because of the distinct substantive content, three composite subscales were generated.

The primary assessment of reliability is reported in Table 2. Inter-item agreement was acceptable for the ITQ scales from the initial administration for type 1 and type 2 participants (alphas = .71/.68 for Diabetes Worries, .85/.84 for Perceptions of Insulin Therapy, .83/.81 for Treatment Satisfaction). The results were similar for the retest administrations (alphas = .75/.70 for Diabetes Worries, .87/.86 for Perceptions of Insulin Therapy, .85/.85 for Treatment Satisfaction).

**Table 2:** Scale Statistics

	T1	T1	T1	T2	T2	T2
**Measure (# of items)**	**Test**	**Retest**	**Test-Retest**	**Test**	**Retest**	**Test-Retest**

**Diabetes Worries (5)**						

Mean^a^	74.0	74.5	p = .348	75.2	76.8	p = .009

SD	16.7	16.9		17.4	16.2	

% max, % min	0.2,7.7	0.6,8.1		0.4,9.0	0.4,9.3	

Reliability^b^	.71	.75	.81	.68	.70	.77

**Perceptions of Insulin Therapy (16)**						

Mean^a^	58.2	56.9	p = .001	57.0	56.4	p = .191

SD	14.9	15.0		15.9	15.9	

% max, % min	0.2,0.0	0.2,0.0		0.2,0.0	0.2,0.7	

Reliability^b^	.85	.87	.90	.84	.86	.85

**Treatment Satisfaction (3)**						

Mean^a^	53.3	54.1	p = .285	52.5	53.6	p = .248

SD	21.4	21.2		23.9	24.6	

% max, % min	2.2,1.6	1.8,1.8		2.8,2.8	2.3,4.6	

Reliability^b^	.83	.85	.83	.81	.85	.74

**Treatment Preference (1)**						

Mean^a^	69.2	66.8	p = .020	58.1	58.3	p = .887

SD	26.0	25.3		30.3	29.0	

% max, % min	3.7,21.0	3.5,16.9		7.4,13.8	6.9,11.6	

Reliability^b^	na	na	.73	na	na	.67

^a ^Test-Retest is the p-value for paired t-test of difference in means across administrations.

^b ^Reliability for each administration is Cronbach's alpha (na = not applicable); Test-Retest reliability is the intraclass correlation coefficient.

Test-retest reliability was assessed in two ways: mean change and correlations across the two pretreatment administrations. Three of the eight tests of change in means were significant (Perceptions of Insulin Therapy and Treatment Preference for type 1 and Diabetes Worries for type 2), but the size of the change was small (less than 0.1 standard deviation units, a measure of effect size). The test-retest associations were acceptable for type 1 and type 2 participants (.81/.77 for Diabetes Worries, .90/.85 for Perceptions of Insulin Therapy, .83/.85 for Treatment Satisfaction, .73/.67 for Treatment Preference).

For the three multi-item scales administered at baseline there was little in the way of floor/ceiling effects; across both pre-treatment administrations in both study populations the percentage of participants with minimum scores ranged from 0.2% to 2.8%, and the percentage with maximum scores ranged from 0% to 9.3%. For the single-item measure minimum scores ranged from 3.5% to 7.4%, and the percentage with maximum scores ranged from 11.6% to 21.0%.

Associations among IITQ scale scores varied within and between populations. Diabetes Worries were not associated with any other score in the type 1 population, but were significantly associated with Treatment Satisfaction and Treatment Preference in the type 2 population (r = .12 and .14, respectively). Associations among other scale scores all were significant and of moderate size (r = .29 to .58, median = .46).

Associations of ITQ measures with subject characteristics are presented in Table 3. The magnitude of all significant correlations was modest. The Diabetes Worries measure was significantly lower for patients who were non-Hispanic White and had higher weight/BMI (type 1 and type 2), and those who had better mental health (type 2 only). The Perceptions of Insulin Therapy measure was significantly higher for patients who were male and older (type 2 only) and who had better mental or physical health (type 1 and type 2). The Treatment Satisfaction measure was significantly higher for patients who were older and had lower weight/BMI and better physical health (type 2 only) and those who had better glucose control (type 1 and type 2). The Treatment Preference measure was significantly higher for patients who had lower weight/BMI (type 2 only) and better mental health (type 1 only).

**Table 3:** Association of IITQ Measures with Participant Characteristics at Baseline

Treatment Outcome	Diabetes Worries	Perceptions of Insulin Therapy	Treatment Satisfaction	Treatment Preference
**Gender (Male = 1)**	-.08/-.05	-.00/.08*	.03/.06	-.01/.01

**Race (Caucasian = 1)**	-.18***/-.10*	.04/.02	.08/.03	.03/.07

**Age (Years)**	-.03/-.04	-.04/.09*	.03/.12**	-.02/.06

**Duration of Diabetes (Years)**	-.05/03	.01/.05	.02/.03	.07/.08

**Weight (Kilograms)**	-.16***/-.16 ***	-.06/-.05	-.00/-.09*	-.05/-.11**

**BMI (kg/m2)**	-.11*/-.09*	-.05/-.07	-.01/-.09*	-.05/-.12**

**A1c (%)**	.00/.01	-.03/-.06	-.14**/-.11**	-.01/-.07

**SF-36 PCS**	-.07/.02	.13**/.14***	.04/.09*	.00/.08

**SF-36 MCS**	-.09/-.12**	.15***/.17***	.08/-.01	.11*/.01

Note: Cell entries are Pearson correlations (type 1/type 2).

* p < .05

** p < .01

*** p < .001

For the Inhaler Performance items (obtained only at follow-up and only from study participants randomized to TI) the scale reliabilities were acceptable for type 1 and type 2 participants (alphas = .86/.90 for Overall, .81/.84 for Device, .86/.88 for Cartridge, .73/.79 for Dosing). Test-retest reliability was not assessed for the device performance measures because these items could not be administered until after patients had used the device. Floor effects were nonexistent for all measures in both type 1 and type 2 populations (% receiving minimum score = 0%). Ceiling effects (type 1/type 2) were minimal to modest for the overall measure and the dose subscale (% receiving maximum score = 4.9%/7.7% and 6.8%/10.6%, respectively) but higher for the device subscale and the cartridge subscale (10.7%/19.1% and 36.1%/39.0%, respectively).

Associations among Inhaler Performance subscale scores were moderate to large (r = .28 to .68, median = .52). Associations with baseline participant characteristics were not assessed because they reflect differential efficacy rather than differences in response patterns (because these measures were obtained after use of the device).

## Discussion

The results of this study provide evidence that the IITQ is a reliable instrument for assessing a broad range of patient reported outcomes (PRO) in patients with type 1 or type 2 diabetes using inhaled insulin. In addition to a measure of diabetes worries, the IITQ also includes measures of perceptions of insulin therapy, treatment system satisfaction, treatment system preference, and inhaler performance.

Including insulin treatment-specific measures in an assessment is important because these measures are likely to be more sensitive to specific but important differences among insulin treatment systems. Device-specific measures are also useful, because device usability may affect adoption of a medication treatment system and persistent use of the system.

Incorporating multiple indicators of treatment system satisfaction and a measure of treatment preference not only allows comparison of the system patients previously used with the one they are currently using, but also provides a prospective measure of overall satisfaction with the current system, interest in continuing to use the system, and willingness to recommend the system to others. Although the IITQ is specifically designed to assess PRO related to insulin therapy, the system satisfaction and system preference items refer to any system for controlling blood glucose levels, so these measures can be used to compare any such treatment systems, including oral agents or injectable agents other than insulin.

The IITQ covers a broader range of outcomes than other instruments designed to assess general diabetes treatment satisfaction (DTSQ) or PRO associated with inhaled insulin therapy [14,15]. The IITQ assesses many of the same elements as the IDSRQ [16], including comfort, convenience, facilitation of self-care, and clinical efficacy, but it is shorter, and it contains items specific to inhaled insulin therapy.

The IITQ has acceptable psychometric properties (internal consistency, test-retest reliability, floor/ceiling effects). Although the test-retest analysis revealed statistically significant changes in means for three of eight tests, the shifts were small, less than 0.1 SD units [17]. Test-retest reliability was not assessed for the device performance measures because this was not possible given the fact that these items could not be administered until after patients had used the device.

IITQ measures were significantly associated with a number of study participant characteristics, though all these associations were of modest magnitude. Given the absence of hypothesized causal connections between IITQ subscales and most correlates, we did not expect strong correlations. We did expect better health status, including SF-36 scores to be associated with lower IITQ worries scores and higher IITQ treatment satisfaction scores. These associations were significant for diabetes worries and SF-36 mental health scores, and for treatment satisfaction and SF-36 physical health scores in patients with type 2 diabetes. We also found more positive perceptions of insulin therapy in individuals with higher SF-36 physical health and mental health scores. These findings correspond to those of a recent study of individuals taking insulin which found that lower levels of general wellbeing and higher levels of diabetes distress were associated with more negative perceptions of insulin therapy [18]. Several studies of insulin naïve individuals have found significant associations between poor wellbeing or distress and negative perceptions of insulin therapy [19-21].

We also found higher levels of treatment satisfaction among those with lower levels of HbA1c. It is not surprising that individuals whose treatment is associated with good blood glucose have positive perceptions of that treatment. We did find one unexpected association between an IITQ measure (diabetes worries) and a measure of health status (BMI): diabetes worries were lower in those with higher BMI. Perhaps lower levels of diabetes worry led to less active diabetes self-care, and consequently to higher BMI.

As expected, we found few significant correlations between IITQ subscale score and other participant characteristics, indicating that IITQ responses are not heavily influenced by extraneous factors, making the IITQ appropriate for multiple populations.

### Study strengths and limitations

Study strengths include the large study populations of individuals with type 1 diabetes and those with type 2 diabetes. Another strength is the standardized instrument administration procedures. Study limitations include the relative lack of racial/ethnic diversity among study participants.

### Research implications

Future research with the IITQ should include confirmatory psychometric analyses in representative populations, and longitudinal studies to assess other psychometric properties of the IITQ, especially the instrument's validity (e.g., construct validity as assessed by known group comparison, criterion validity as assessed by reference to a gold standard measure, and predictive validity as assessed by subsequent continuation of the study medication). Of special interest will be studies that assess objective clinical outcomes and patient perception measures that predict treatment satisfaction and treatment preference; the benefits of such analyses are described elsewhere [22,23].

## Conclusions

In summary, this study provides evidence that the IITQ is a reliable instrument for assessing a broad range of patient reported outcomes in individuals with type 1 or type 2 diabetes, especially those taking inhaled insulin. Future research should further assess this instrument's validity and its suitability for other research situations.

## Competing interests

The authors receive compensation for consultation services provided to MannKind Corporation. This study was funded by an unrestricted grant to the authors from MannKind Corporation. The authors are responsible for all aspects of manuscript preparation and submission. No medical writers were involved in preparation of this manuscript.

## Authors' contributions

RRR and MP conceived of the study, participated in the design of the study, and drafted the manuscript. MP performed the statistical analysis. RRR and MP read and approved the final manuscript.

## Supplementary Material

Additional file 1 Appendix A: IITQ Click here for file

Additional file 2 Appendix B: IITQ Scoring Instructions Click here for file
