# Supplementary material for: Psychometric properties of an instrument for assessing the experience of patients treated with inhaled insulin: the Inhaled Insulin Treatment Questionnaire (IITQ)
Source: Health Qual Life Outcomes. 2010 Mar 24;8:32. doi: 10.1186/1477-7525-8-32 (PMC2856530; doi:10.1186/1477-7525-8-32)
Supplement: Additional file 1 — Appendix A: IITQ [file 1477-7525-8-32-S1.DOC]

**Appendix A: IITQ**

**The following questions are about YOUR CURRENT experience.**

**Please answer every question. Some questions may look like others, but each one is different. Please take the time to read and answer each question carefully, and circle the answer that best describes your opinion. *Thank you for completing this survey!***

| **1. I worry about high blood sugar levels.** | | | | | |
| --- | --- | --- | --- | --- | --- |
| 1  strongly disagree | 2  disagree | 3  mildly disagree | 4  mildly agree | 5  agree | 6  strongly agree |

| **2. I worry about low blood sugar levels.** | | | | | |
| --- | --- | --- | --- | --- | --- |
| 1  strongly disagree | 2  disagree | 3  mildly disagree | 4  mildly agree | 5  agree | 6  strongly agree |

| **3. I worry about unpredictable blood sugar levels.** | | | | | |
| --- | --- | --- | --- | --- | --- |
| 1  strongly disagree | 2  disagree | 3  mildly disagree | 4  mildly agree | 5  agree | 6  strongly agree |

| **4. I worry about high blood sugar levels after meals.** | | | | | |
| --- | --- | --- | --- | --- | --- |
| 1  strongly disagree | 2  disagree | 3  mildly disagree | 4  mildly agree | 5  agree | 6  strongly agree |

| **5. I worry about diabetes complications.** | | | | | |
| --- | --- | --- | --- | --- | --- |
| 1  strongly disagree | 2  disagree | 3  mildly disagree | 4  mildly agree | 5  agree | 6  strongly agree |

*Please answer these questions in terms of your CURRENT mealtime insulin.*

| **6. Taking insulin is convenient.** | | | | | |
| --- | --- | --- | --- | --- | --- |
| 1  strongly disagree | 2  disagree | 3  mildly disagree | 4  mildly agree | 5  agree | 6  strongly agree |

| **7. Taking insulin when I am away from home is difficult.** | | | | | |
| --- | --- | --- | --- | --- | --- |
| 1  strongly disagree | 2  disagree | 3  mildly disagree | 4  mildly agree | 5  agree | 6  strongly agree |

| **8. Taking insulin is comfortable.** | | | | | |
| --- | --- | --- | --- | --- | --- |
| 1  strongly disagree | 2  disagree | 3  mildly disagree | 4  mildly agree | 5  agree | 6  strongly agree |

| **9. Taking insulin is complicated.** | | | | | |
| --- | --- | --- | --- | --- | --- |
| 1  strongly disagree | 2  disagree | 3  mildly disagree | 4  mildly agree | 5  agree | 6  strongly agree |

| **10. Taking insulin is time-consuming.** | | | | | |
| --- | --- | --- | --- | --- | --- |
| 1  strongly disagree | 2  disagree | 3  mildly disagree | 4  mildly agree | 5  agree | 6  strongly agree |

| **11. Taking insulin is easy.** | | | | | |
| --- | --- | --- | --- | --- | --- |
| 1  strongly disagree | 2  disagree | 3  mildly disagree | 4  mildly agree | 5  agree | 6  strongly agree |

| **12. Taking the right amount of insulin is difficult.** | | | | | |
| --- | --- | --- | --- | --- | --- |
| 1  strongly disagree | 2  disagree | 3  mildly disagree | 4  mildly agree | 5  agree | 6  strongly agree |

| **13. Taking every dose of insulin is easy.** | | | | | |
| --- | --- | --- | --- | --- | --- |
| 1  strongly disagree | 2  disagree | 3  mildly disagree | 4  mildly agree | 5  agree | 6  strongly agree |

| **14. Taking insulin at the right time is difficult.** | | | | | |
| --- | --- | --- | --- | --- | --- |
| 1  strongly disagree | 2  disagree | 3  mildly disagree | 4  mildly agree | 5  agree | 6  strongly agree |

| **15. Taking insulin when I travel is difficult.** | | | | | |
| --- | --- | --- | --- | --- | --- |
| 1  strongly disagree | 2  disagree | 3  mildly disagree | 4  mildly agree | 5  agree | 6  strongly agree |

| **16. Taking insulin when I am away from home is embarrassing.** | | | | | |
| --- | --- | --- | --- | --- | --- |
| 1  strongly disagree | 2  disagree | 3  mildly disagree | 4  mildly agree | 5  agree | 6  strongly agree |

| **17. Taking insulin is painful.** | | | | | |
| --- | --- | --- | --- | --- | --- |
| 1  strongly disagree | 2  disagree | 3  mildly disagree | 4  mildly agree | 5  agree | 6  strongly agree |

| **18. Taking insulin helps me control my blood glucose.** | | | | | |
| --- | --- | --- | --- | --- | --- |
| 1  strongly  disagree | 2  disagree | 3  mildly disagree | 4  mildly agree | 5  agree | 6  strongly agree |

| **19. Taking insulin helps me avoid high blood glucose levels.** | | | | | |
| --- | --- | --- | --- | --- | --- |
| 1  strongly disagree | 2  disagree | 3  mildly disagree | 4  mildly agree | 5  agree | 6  strongly agree |

| **20. Taking insulin helps me avoid low blood glucose levels.** | | | | | |
| --- | --- | --- | --- | --- | --- |
| 1  strongly disagree | 2  disagree | 3  mildly disagree | 4  mildly agree | 5  agree | 6  strongly agree |

| **21. Overall I am satisfied taking insulin.** | | | | | |
| --- | --- | --- | --- | --- | --- |
| 1  strongly disagree | 2  disagree | 3  mildly disagree | 4  mildly agree | 5  agree | 6  strongly agree |

*The following questions are about the system you CURRENTLY use to control your blood glucose levels. This system includes the medications you are taking (pills and insulin), the amount of medication you take, your schedule for taking the medication, and the way you take the medication.*

| **22. Overall I am satisfied with the system I currently use to control my blood glucose levels.** | | | | | |
| --- | --- | --- | --- | --- | --- |
| 1  strongly disagree | 2  disagree | 3  mildly disagree | 4  mildly agree | 5  agree | 6  strongly agree |

| **23. I would like to continue using the system I currently use to control my blood glucose levels.** | | | | | |
| --- | --- | --- | --- | --- | --- |
| 1  strongly disagree | 2  disagree | 3  mildly disagree | 4  mildly agree | 5  agree | 6  strongly agree |

| **24. I would recommend the system I currently use to control my blood glucose levels to others.** | | | | | |
| --- | --- | --- | --- | --- | --- |
| 1  strongly disagree | 2  disagree | 3  mildly disagree | 4  mildly agree | 5  agree | 6  strongly agree |

| **25. I prefer the system I currently use to control my blood glucose levels to my previous system.** | | | | | |
| --- | --- | --- | --- | --- | --- |
| 1  strongly disagree | 2  disagree | 3  mildly disagree | 4  mildly agree | 5  agree | 6  strongly agree |

*The following questions refer to the device that you CURRENTLY use to take insulin. Please answer these questions in terms of your CURRENT device.*

| **26. Learning to use the inhaler is easy.** | | | | | |
| --- | --- | --- | --- | --- | --- |
| 1  strongly disagree | 2  disagree | 3  mildly disagree | 4  mildly agree | 5  agree | 6  strongly agree |

| **27. Using the inhaler is easy.** | | | | | |
| --- | --- | --- | --- | --- | --- |
| 1  strongly disagree | 2  disagree | 3  mildly disagree | 4  mildly agree | 5  agree | 6  strongly agree |

| **28. Inhaling through the inhaler is comfortable.** | | | | | |
| --- | --- | --- | --- | --- | --- |
| 1  strongly disagree | 2  disagree | 3  mildly disagree | 4  mildly agree | 5  agree | 6  strongly agree |

| **29. Knowing I have reached the proper inhalation rate is easy.** | | | | | |
| --- | --- | --- | --- | --- | --- |
| 1  strongly disagree | 2  disagree | 3  mildly disagree | 4  mildly agree | 5  agree | 6  strongly agree |

| **30. It is easy to insert the cartridge into the inhaler.** | | | | | |
| --- | --- | --- | --- | --- | --- |
| 1  strongly disagree | 2  disagree | 3  mildly disagree | 4  mildly agree | 5  agree | 6  strongly agree |

| **31. It is easy to tell that the inhaler cartridge is properly inserted.** | | | | | |
| --- | --- | --- | --- | --- | --- |
| 1  strongly disagree | 2  disagree | 3  mildly disagree | 4  mildly agree | 5  agree | 6  strongly agree |

| **32. It is easy to remove the cartridge from the inhaler.** | | | | | |
| --- | --- | --- | --- | --- | --- |
| 1  strongly disagree | 2  disagree | 3  mildly disagree | 4  mildly agree | 5  agree | 6  strongly agree |

| **33. It is easy to keep the inhaler clean.** | | | | | |
| --- | --- | --- | --- | --- | --- |
| 1  strongly disagree | 2  disagree | 3  mildly disagree | 4  mildly agree | 5  agree | 6  strongly agree |

| **34. The inhaler is convenient to use.** | | | | | |
| --- | --- | --- | --- | --- | --- |
| 1  strongly disagree | 2  disagree | 3  mildly disagree | 4  mildly agree | 5  agree | 6  strongly agree |

| **35. I feel confident that I have inhaled the proper dose.** | | | | | |
| --- | --- | --- | --- | --- | --- |
| 1  strongly disagree | 2  disagree | 3  mildly disagree | 4  mildly agree | 5  agree | 6  strongly agree |
