# Supplementary material for: Psychometric properties of an instrument for assessing the experience of patients treated with inhaled insulin: the Inhaled Insulin Treatment Questionnaire (IITQ)
Source: Health Qual Life Outcomes. 2010 Mar 24;8:32. doi: 10.1186/1477-7525-8-32 (PMC2856530; doi:10.1186/1477-7525-8-32)
Supplement: Additional file 2 — Appendix B: IITQ Scoring Instructions [file 1477-7525-8-32-S2.DOC]

**Appendix B: IITQ Scoring Instructions**

This document provides scoring instructions for the Inhaled Insulin Treatment Questionnaire (IITQ). This includes the recoding of numerical responses printed on the questionnaire (with reverse coding where appropriate) and the calculation of scale scores.

| **Questionnaire response scoring**  **(all items)** | **Conversion to scale scoring (all items)** |
| --- | --- |
| 1 strongly disagree | 0 |
| 2 disagree | 20 |
| 3 mildly disagree | 40 |
| 4 mildly agree | 60 |
| 5 agree | 80 |
| 6 strongly agree | 100 |
| Blank or multiple responses | missing |

Several items (7,9,10,12,14,15,16,17) need to have the scale scoring reversed so that the direction of scoring (higher/lower, positive/negative) within a measure is consistent.

| **Scale scoring**  **(items 7,9,10,12,14,15,16,17)** | **Reversed scale scoring**  **(items 7,9,10,12,14,15,16,17)** |
| --- | --- |
| 0 | 100 |
| 20 | 80 |
| 40 | 60 |
| 60 | 40 |
| 80 | 20 |
| 100 | 0 |
| missing | missing |

All scale scores are computed as the mean of the scale scored items (with reverse scoring as necessary).

Diabetes Worries: items 1 through 5

Perceptions of Insulin Treatment: items 6 through 21

Treatment System Satisfaction: items 22 through 24

Treatment System Preference: item 25

Inhaler Performance – Overall: items 26 through 35

Inhaler Performance – Device: items 26,27,28,33,34

Inhaler Performance – Dose: items 29,35

Inhaler Performance – Device: items 30 through 32
